# 16S rRNA Sequencing Reveals Relationship Between Potent Cellulolytic Genera and Feed Efficiency in the Rumen of Bulls

**DOI:** 10.3389/fmicb.2018.01842

**Published:** 2018-08-10

**Authors:** Emily McGovern, David A. Kenny, Matthew S. McCabe, Claire Fitzsimons, Mark McGee, Alan K. Kelly, Sinéad M. Waters

**Affiliations:** ^1^Teagasc, Animal and Bioscience Research Department, Animal & Grassland Research and Innovation Centre, Teagasc, Grange, Dunsany, Ireland; ^2^UCD College of Health and Agricultural Sciences, University College Dublin, Belfield, Ireland; ^3^Teagasc, Livestock Research Systems Department, Animal & Grassland Research and Innovation Centre, Teagasc, Grange, Dunsany, Ireland

**Keywords:** rumen, microbiota, phylogenetic analysis, feed efficiency, nutrition

## Abstract

The rumen microbial population dictates the host’s feed degradation capacity and subsequent nutrient supply. The rising global human population and intensifying demand for animal protein is creating environmental challenges. As a consequence, there is an increasing requirement for livestock with enhanced nutrient utilization capacity in order to more efficiently convert plant material to high quality edible muscle. In the current study, residual feed intake (RFI), a widely used and a highly accepted measure of feed efficiency in cattle, was calculated for a combination of three cohorts of Simmental bulls. All animals were managed similarly from birth and offered concentrate *ad libitum* in addition to 3 kg of grass silage daily during the finishing period. Solid and liquid rumen digesta samples collected at slaughter and were analyzed using amplicon sequencing targeting the *16S rRNA* gene utilizing the Illumina MiSeq platform. Volatile fatty acid analysis was also conducted on the liquid digesta samples. Spearman’s correlation coefficient was utilized to determine the association between RFI and bacterial and archaeal taxa and inter-taxonomic relationships. The data indicate a tendency toward an increase in butyrate (*P* = 0.06), which corresponds with an increase in plasma β-hydroxybutyrate concentration in low RFI (LRFI) bulls in comparison to their high RFI (HRFI) contemporaries (*P* < 0.05). A decrease in propionate (*P* < 0.05) was also recorded in the rumen of LRFI in comparison to HRFI bulls. These results indicate alternate fermentation patterns in the rumen of LRFI bulls. The data also identified that OTUs within the phyla *Tenericutes, Fibrobacteres*, and *Cyanobacteria* may potentially influence RFI phenotype. In particular, a negative association between *F. succinogenes* and RFI was evident. The unique cellulolytic metabolism of *F. succinogenes* suggests it could contribute to host efficiency by providing substrate to the host ruminant and other microbial populations (e.g., *Selenomonas ruminantium, Methanobrevibacter*, and *Methanomassiliicoccaceae)* in the rumen. This study provides evidence that bacterial OTUs within common phyla could influence ruminant feed efficiency phenotype through their role in ruminal degradation of complex plant polysaccharides or increased capability to harvest nutrients from ingested feed.

## Introduction

The world’s population is estimated to reach 9.15 billion by 2050 (Opio et al., 2013). This will place increasing demands, in the region of an additional 70%, on food production systems necessitating additional crop availability for animal feed to support the ever growing human requirement for meat and milk production (Alexandratos and Bruinsma, 2012). Profitability of beef production systems relies on achieving maximum outputs using minimum inputs (Ahola and Hill, 2012). Given that feed accounts for the single largest variable cost in ruminant production systems (Finneran et al., 2010) global interest has focused on the identification of cattle that are more feed efficient (Ahola and Hill, 2012). Improving feed efficiency in the livestock sector will be crucial for sustainable animal production as it has the potential to amplify nutrient utilization from feed, improve profitability and reduce agricultural greenhouse gas emissions (Crowley et al., 2010). Residual feed intake (RFI) is a measure of feed efficiency, it is defined as the difference between observed and predicted feed intake calculated as the residuals from a multiple regression model of intake on the various energy expenditures (e.g., weight and growth rate) (Koch et al., 1963). It is the preferred measure of feed efficiency due to its independence from production traits, thus, inter-animal differences in RFI is most likely as a result of variation in component metabolic processes rather than variation due to differences at the production level (Nkrumah et al., 2006).

Ruminants rely on the microbiota (bacteria, archaea, ciliate protozoa, fungi, and viruses) present in the rumen to degrade their feed and produce volatile fatty acids (VFAs), microbial proteins and vitamins. The primary VFAs produced in the rumen, acetate, propionate, and butyrate, provide up to 70% of the host’s energy requirements (Flint et al., 2008). Animal performance is dependent on numerous factors including genotype, stage of development and the chemical composition and quantity of feed offered (Carberry et al., 2012; Keogh et al., 2015; McDonnell et al., 2016; Shabat et al., 2016), which also influence the rumen microbiome (Carberry et al., 2012; Jami et al., 2013; Shabat et al., 2016; McGovern et al., 2017). Therefore there is potential for a link between feed efficiency and microbiota present in the rumen (Ahola and Hill, 2012). Research from our own group and others have indeed identified links between rumen microbiome and inter-animal variation in feed efficiency phenotype (Carberry et al., 2012; Jewell et al., 2015; Myer et al., 2015; Roehe et al., 2016; Shabat et al., 2016; Li and Guan, 2017). Shabat et al. (2016) previously reported rumen microbiome genes and species that could predict (91% accuracy) of the variation in an animal’s feed efficiency phenotype and discerned that reduced rumen microbial community diversity may support a more feed efficient animal.

Although research investigating the role of the rumen microbiome in influencing feed efficiency phenotype exists, data are limited, especially for beef cattle. Furthermore, many of these studies are restricted to only investigating rumen liquid digesta and use alternate methods of measuring feed efficiency, such as feed conversion ratio (FCR), which can be problematic due to the unfavorable genetic correlations with between growth rate and mature body size (Schenkel et al., 2004). This implies that selection of cattle for improved FCR will lead to an increase in mature body size and therefore, maintenance requirements (Schenkel et al., 2004; Crews, 2005). This has negative economic and environmental ramifications for beef production (Arthur et al., 2001; Schenkel et al., 2004; Crews, 2005).

In the current study, RFI phenotype was determined for a combination of four cohorts of Simmental bulls over 4 years. Archaeal and bacterial populations present in both the rumen solid and liquid digesta of Simmental bulls divergent for RFI phenotype were determined using amplicon sequencing targeting the *16S rRNA* gene. Rumen fluid from the bulls was further analyzed to establish its metabolomic composition. Differences in microbial populations and metabolic profiles were examined between divergent phenotypes. Archaeal and bacterial populations were correlated to RFI phenotype as well as inter-taxonomically, in order to investigate possible microbial drivers of feed efficiency. As data were derived from a combined population, there was increased strength in observed association due to the conservation of the relationship between the cohorts. Our data address gaps in knowledge surrounding the rumen microbial ecosystem and provides evidence of potential mechanisms relating components of the rumen microbiome to host feed efficiency phenotype.

## Materials and Methods

All procedures involving animals from years 1–3, in this study were conducted under an experimental license from the Irish Department of Health and Children, while procedures involving animals from year four were conducted under an experimental license (AE19132/P011) from the Health Products Regulatory Authority. Both licenses where issued in accordance with the cruelty to Animals Act 1876 and the European Communities (Amendment of Cruelty to Animals Act 1876) Regulations 2002 and 2005.

### Animal Model

A total of 87 Simmental bulls over 4 years comprising 20, 33, 14, and 20 bulls in years 1, 2, 3, and 4, respectively, were used. They were the progeny of spring-calving cows bred to Simmental sires as described by Lawrence et al. (2011). The RFI measurement period was carried out during the finishing phase. Animals were offered *ad libitum* concentrates (860 g/kg rolled barley, 60 g/kg soya bean meal, 60 g/kg molasses, and 20 g/kg minerals per vitamins) and 3 kg (dry matter = 242g/kg) of grass silage (Fitzsimons et al., 2014; McKenna et al., unpublished) for the duration of the trial period and prior to slaughter. All animals had continuous access to clean fresh drinking water. Individual feed intake was recorded daily using electronically controlled Calan gates (American Calan Inc., Northwood, NH, United States). The RFI measurement period lasted, on average, for 97 days (101, 107, 108, and 70 days in years 1, 2, 3, and 4, respectively). Mean age at the start of the RFI measurement period was 425 days (*SD* = 39.8). At the end of each period animals were slaughtered at a mean bodyweight of 580 (*SD* = 74.1) kg.

### RFI Calculation

Residual feed intake was calculated for each animal as the difference between actual dry matter intake (DMI) and expected DMI. Expected DMI was computed for each animal using a multiple regression model, regressing DMI on metabolic body weight (MBW) and average daily live weight gain (ADG), with year included as a class variable. The model used was:

Yj=β0+τi+β1MBWj+β2ADGj+β3 BFj+ej

Where Y*j* is the average DMI of the *j*th animal, β0 is the partial regression intercept, β1 is the partial regression coefficient on MBW^0.75^, β2 is the partial regression coefficient on ADG, and e*j* is the uncontrolled error of the *j*th animal. The coefficient of determination (R^2^) from this model was equal to 0.7 (*P* < 0.001) and the model was subsequently used to predict DMI for each animal.

Rumen solid and liquid samples from cattle for the top (mean RFI = 0.85) and bottom (mean RFI = -0.76) sextiles were subjected to microbiomic analysis (*n* = 60). No bulls from year three ranked in upper sextiles for RFI. Mean slaughter age for LRFI and HRFI bulls was 515.4 days (*SD* = 42.3) and 525.5 days (*SD* = 33.6), respectively. There was no statistical difference in age between treatment groups (*P* > 0.05). For logistical reasons seven samples could not be obtained during the sampling process; leaving a sample size of *n* = 53; 12 solid LRFI, 14 solid HRFI, 13 liquid LRFI and 14 liquid HRFI for microbiomic analysis.

### Sample Collection at Slaughter

Blood samples were obtained by jugular venepuncture from each animal before feeding, on the day prior to slaughter. Blood samples were collected into a 9- and 4-ml evacuated tube containing lithium heparin and sodium fluoride – EDTA K3, respectively, as anticoagulants (Greiner Vacuette, Cruinn Diagnostics, Dublin, Ireland).

Digesta contents were sampled from five different locations, inclusive of the dorsal and ventral sacs, within the rumen of each bull immediately after slaughter. Liquid and solid fractions of rumen content were separated by squeezing contents through four layers of cheese cloth. Both fractions were immediately frozen after separation in liquid nitrogen and then stored at -80°C.

### Metabolite Analysis

#### Rumen Metabolites

Rumen fluid (20 ml) of bulls from year one and two, were preserved with 0.5 mL of 9 M sulphuric acid and stored at –20°C for subsequent analysis VFAs. These samples were analyzed using an automated gas chromatograph (Shimadzu Gas Chromatography GC-8A, Shimadzu Corporation, Kyoto, Japan; Brotz and Schaefer, 1987) as previously described by Owens et al. (2008). Rumen fluid samples from year four were snap frozen and stored at –20°C for preservation. VFA analysis was conducted using model 3800 Varian gas chromatograph as previously described by Lynch et al. (2007). Absolute concentrations of VFAs for each sample were converted to molar proportions (*n* = 29, no result was obtained for one sample).

Lactic acid concentration was determined using the SP-Ace Clinical Chemical Analyzer, (Alfa Wassermann Inc., West Caldwell, NJ, United States) and the l-lactic acid UV-method test kit (Roche/R-Biopharm, Darmstadt, Germany), whereas D-lactate concentration was determined using the enzyme D-lactate dehydrogenase (Roche/R-Biopharm). The concentration of ammonia was determined using the SP-Ace Clinical Chemical Analyzer and the Thermo Electron Infinity ammonia liquid stable reagent kinetic method (Thermo Fisher Scientific Inc., Waltham, MA, United States).

#### Blood Metabolite

Concentrations of β-hydroxybutyrate (βHB) was determined according to Lawrence et al. (2011). All metabolite concentrations were measured on an automatic analyser (AU400; Olympus, Tokyo, Japan). The sensitivity of each assay was defined as the lowest concentration detectable.

### DNA Extraction

Approximately 20 g of frozen rumen liquid and solid digesta sample from each of the animals was used as a representative sample (McCabe et al., 2015). Each sample was homogenized to a fine frozen powder under liquid nitrogen using a pestle and mortar and stored at -80°C. Approximately 250 mg of homogenized frozen powder was used for DNA extraction from the solid and liquid fractions. DNA was extracted using the repeated bead beating and column purification method (Yu and Morrison, 2004). DNA quality was assessed on an agarose gel. DNA extract yield and purity were assessed with two consecutive readings on the Nanodrop 1000 spectrophotometer. The average A_260_/A_280_ and A_260_/A_230_ ratio for samples were 1.87 and 2.24, respectively.

### 16S rRNA Amplicon Library Preparation

Amplicon libraries (*n* = 53) targeting the V4 region of the 16S rRNA gene in bacteria and archaea were generated by PCR amplification. PCR reaction was performed for amplicon libraries with 20 ng of rumen microbial DNA from solid and liquid fractions and 515F forward and 806R reverse primers (Caporaso et al., 2011) designed with Nextera overhang adapters, using 1X KAPA HiFi HotStart ReadyMix DNA polymerase (Roche Diagnostics, West Sussex, United Kingdom). Cycle conditions were 95°C for 3 min, 28 PCR cycles; 95°C for 30 s, 55°C for 30 s, 72°C for 30 s and then 72°C for 5 min. Amplicons were purified using QIAquick PCR Purification Kit (Qiagen, Manchester, United Kingdom). A second PCR step attached dual indices and Illumina sequencing adapters using Nextera XT index kit. Cycle conditions were 95°C for 3 min, 8 PCR cycles; 95°C for 30 s, 55°C for 30 s, 72°C for 30 s and then 72°C for 5 min. Amplicon generation was validated through visualization on a 2% (w/v) agarose gel and concentration was measured on the Nanodrop 1000 spectrophotometer. Amplicons were pooled in equal concentrations and gel purified to remove unwanted products using the Qiagen Gel Extraction Kit (Qiagen, Manchester, United Kingdom). An extra purification step using the QIAquick Purification Kit (Qiagen, Manchester, United Kingdom) was performed to remove residual agarose. The pooled purified libraries were measured for purity and quantity on the Nanodrop 1000 and further quantified using the KAPA SYBR FAST universal kit with Illumina Primer Premix (Roche Diagnostics, West Sussex, United Kingdom). The library pool was then diluted and denatured as recommended by Illumina MiSeq library preparation guide. The cycle was conducted using 500 cycle MiSeq reagent kits.

### Sequence Analysis

At this point, four samples were removed from the analysis due to low sequence output; 2 LRFI solid and 2 HRFI solid (*n* = 49). 16S rRNA gene amplicon sequences were quality checked with FASTQC (Andrews, 2010) and overlapping reads were merged using BBMerge from the BBMap package^1^. Merged reads from all samples were combined into a single dataset for processing with QIIME (version 1.9). Chimeric sequences were removed via USEARCH using the ChimeraSlayer GOLD database (Edgar et al., 2011). A combination of *de novo* and reference based OTU identification was carried out using the open reference calling method implemented within QIIME. Sequences were clustered at 97% identity into individual OTUs using UCLUST (Edgar et al., 2011) and a single representative sequence from each clustered OTU was aligned to the Greengenes database (version gg_13_8). Taxonomic classification for each OTU was determined using the default classifier; UCLUST (Edgar et al., 2011) within QIIME. OTUs with fewer than 100 sequences across all samples were excluded from further analysis (McMurdie and Holmes, 2014). A rarefaction curve was constructed to ensure sufficient sequencing depth had been achieved (**Supplementary Figure S1**). Sequence files associated with each sample have been submitted to the NCBI Sequence Read Archive (Accession no. PRJNA472866).

A basic local alignment search tool (BLAST) against the NCBI non-redundant nucleotide data (Morgulis et al., 2008; Edgar, 2010) was used to further classify methanogens representative OTU sequences of interest.

### Statistical Analysis

Raw sequencing counts were used to compute relative abundance per taxonomic level and OTU tables. A Wilcoxon rank sum test with correction for multiple testing using the Benjamini Hochberg method was then used to identify individual taxa differently represented across treatment groups. A transformation-based principal component analysis was performed by square root transformation of the relative abundance data and subjecting Bray Curtis similarity analysis. Distance based permutation multivariate analysis of variance (PERMANOVA) (Anderson and Walsh, 2013) was performed to test the null hypothesis that there were no differences in the microbial community structure across treatment at a significance level of *P* = 0.05 based on 9,999 permutations. Alpha diversity (observed OTUs, richness, Shannon and Simpson diversity indices) were calculated for each sample in PRIMER7. Group means were calculated for alpha diversity, VFA concentrations, daily DMI, RFI, and ADG within treatment groups. A Student’s *T*-test was used to analyze differences between means. Spearman’s correlation coefficient was used to examine if any significant correlations existed between RFI coefficients and the taxon present in the rumen and selected inter-taxonomic relationships. Inter-taxonomic relationships were investigated at genus level due to the limited sequence resolution available with the Illumina MiSeq. Spearman’s correlation analysis was conducted using Hmisc package R Studio (v3.1). For this analysis relationships were only explored if there was more than 0.1%-fold change of microbial taxa between the treatment groups.

## Results

In total, 30 Simmental bulls divergent in RFI ranking (*n* = 15 HRFI and *n* = 15 LRFI) were used in this study (*P* < 0.0001) (**Table 1**). There was no interaction between RFI and year or individual microbial taxa and RFI (*P* > 0.05). HRFI bulls had an increased DMI in comparison to LRFI animal and consumed 23.5% more than their LRFI counterparts, respectively (*P* < 0.001) (**Table 1**). ADG and MBW did not differ between the treatment groups (*P* > 0.05) (**Table 1**).

**Table 1:** Dry matter intake (DMI) of concentrate feed, residual feed intake (RFI), and average daily gain (ADG) (kg) of bulls ranked low (LRFI) and high RFI (HRFI).

	HRFI	*SD*	LRFI	*SD*	*P-*value
**HRFI *n* = 15 LRFI *n* = 15**					
DMI (kg/d)	10.18	1.37	8.24	0.94	<0.01
RFI	0.85	0.48	-0.76	0.23	<0.01
ADG (kg)	1.69	0.35	1.57	0.41	NS

*P-values are derived using a Student’s T-test to assess the differences between treatments.*

The Illumina Miseq amplicon sequencing generated 16,711,973 total reads giving an average of 168,807.8 ± 57,658 reads per sample. This reduced to 6,281,459 when sequences were merged and quality filtered. The average number of counts per sample that were assigned to an OTU (post filtering) was 123,729 ± 45,617.

### Metabolite Analysis

Volatile fatty acid analysis revealed an increase in the molar proportion of propionic acid in the rumen of HRFI in comparison to LRFI bulls (*P* < 0.05) (**Table 2**). The molar proportion of butyric acid tended to be increased in LRFI animals in comparison to HRFI animals (*P* = 0.06) (**Table 2**). In correspondence to this βHB was increased in LRFI (0.25 ± 0.09) bulls in comparison to HRFI (0.18 ± 0.05) bulls (*P* < 0.01). There was no difference in the molar proportion of acetic, valeric, isobutyric iso-valeric acid or concentrations of total VFAs, lactate, or ammonia. Acetate:Propionate (A:P) ratio was increased in LRFI bulls in comparison to HRFI bulls (*P* < 0.05) (**Table 2**).

**Table 2:** Mean molar proportions and standard deviation (SD) of individual rumen volatile fatty acids (VFA) and mean concentration and SD of total VFAs, lactate and ammonia for high RFI (HRFI) and low RFI (LRFI) bulls.

	HRFI	*SD*	LRFI	*SD*	*P-*value
**HRFI *n* = 15 LRFI *n* = 14**					
Acetic	0.52	0.07	0.56	0.06	NS
Propionic	0.32	0.07	0.26	0.08	<0.05
A:P	1.78	0.70	2.45	0.94	<0.05
Iso-butyric	0.01	0.01	0.02	0.01	NS
Butyric	0.10	0.02	0.13	0.04	<0.1
Iso-valeric	0.10	0.07	0.02	0.01	NS
Valeric	0.04	0.01	0.04	0.02	NS
Total VFA (mmol/L)	115.9	27.9	109.9	28	NS
Ammonia (g/L)	20.70	14.00	41.70	51.90	NS
Lactate (ng/100)	0.20	0.26	0.26	0.32	NS

*P-values are derived using a Student’s *T*-test to assess the differences between treatments.*

### Ordination Analysis of Microbial Community Structure

There was no difference in community diversity between solid and liquid digesta phases (*P* > 0.05). No difference in community diversity was observed between LRFI and HRFI bulls in either solid or liquid digesta for archaea or bacteria (*P* > 0.05). Alpha diversity indicators; species presence, individual OTUs present, species richness, species evenness and *alpha* diversity matrices (Shannon, Simpson), showed no difference between HRFI and LRFI treatments for archaea or bacteria for either liquid (**Table 3**) or solid (**Table 4**) ruminal phases (*P* > 0.05).

**Table 3:** Effect of high RFI (HRFI) and low RFI (LRFI) phenotype on species presence, individual OTUs present, species richness, species evenness, and alpha diversity matrices (Shannon, Simpson) in the rumen liquid phase.

	Liquid				
	HRFI	*SD*	LRFI	*SD*	*P-*value
**HRFI *n* = 14 LRFI *n* = 13**					
Species	2226	570	2083	538	NS
Individuals	120001	50708	105909	44989	NS
Richness	190.58	43.19	180.47	40.82	NS
Evenness	0.57	0.41	0.60	0.07	NS
Shannon	4.35	0.41	4.53	0.56	NS
Simpson	0.94	0.03	0.94	0.03	NS

*P-values are derived using a Student’s T-test to assess the differences between treatments.*

**Table 4:** Effect of high RFI (HRFI) and low RFI (LRFI) phenotype on species presence, individual OTUs present, species richness, species evenness, and alpha diversity matrices (Shannon, Simpson) in the rumen solid phase.

	Solid				
	HRFI	*SD*	LRFI	*SD*	*P-*value
**HRFI *n* = 13 LRFI *n* = 11**					
Species	2440	446	2581	712	NS
Individuals	124701	26591	150851	50879	NS
Richness	207.91	35.54	216.29	53.79	NS
Evenness	0.63	0.05	0.63	0.06	NS
Shannon	4.89	0.45	4.96	0.51	NS
Simpson	0.97	0.02	0.97	0.02	NS

*P-values are derived using a Student’s T-test to assess the differences between treatments.*

### Effect of RFI Phenotype on the Archaeal and Bacterial Populations

Nineteen phyla were classified in the rumen of the Simmental bulls. *Bacteroidetes* and *Firmicutes* were the dominant phyla present in both the solid and liquid phase of the rumen, and did not differ between HRFI and LRFI (*P* > 0.05) (**Tables 5**, **6**). The ratio of *Firmicutes:Bacteroidete* (F:B) was not affected by RFI phenotype (*P* > 0.05) in either the rumen liquid (**Table 5**) or solid digesta (**Table 6**). The next most dominant phyla in both phases were *Proteobacteria* and *Euryarchaeota*. They did not differ between feed efficiency treatments (*P* > 0.05) (**Tables 5**, **6**). The phyla *Fibrobacteres* was increased by 21% in LRFI in comparison to HRFI bulls in the liquid phase (*P* < 0.1) and 26% in LRFI in comparison to HRFI bulls in the solid phase. However, this was not significant after false discovery rate (FDR) correction (**Tables 5**, **6**).

**Table 5:** Mean relative abundance of phyla classified in the rumen liquid fraction of Simmental bulls phenotypically divergent for residual feed intake (RFI).

	Liquid						
Phylum	HRFI	LRFI	*P-*value	FDR	Log2 LRFI/HRFI	ρ	*P-*value
**HRFI *n* = 14 LRFI *n* = 13**						
F:B	1.05	1.00	NS	NS	n/a	0.15	NS
*Actinobacteria*	1.10	1.53	NS	NS	0.48	0.10	NS
*Bacteroidetes*	34.31	35.09	NS	NS	0.03	-0.09	NS
*Chloroflexi*	0.17	0.21	NS	NS	0.32	-0.14	NS
*Cyanobacteria*	0.71	1.11	<0.1	NS	0.65	-0.44	<0.05
*Elusimicrobia*	0.06	0.14	NS	NS	1.15	-0.27	NS
*Euryarchaeota*	2.94	3.65	NS	NS	0.31	-0.14	NS
*Fibrobacteres*	0.14	0.67	<0.01	NS	2.24	-0.58	<0.01
*Firmicutes*	36.04	34.95	NS	NS	-0.04	0.26	NS
*LD1*	0.00	0.00	NS	NS	-3.34	0.08	NS
*Lentisphaerae*	0.06	0.04	NS	NS	-0.52	-0.01	NS
*Planctomycetes*	0.17	0.20	NS	NS	0.28	0.07	NS
*Proteobacteria*	21.03	18.63	NS	NS	-0.17	0.03	NS
*Spirochaetes*	0.96	0.73	NS	NS	-0.39	-0.06	NS
*SR1*	0.01	0.01	NS	NS	0.68	-0.07	NS
*Synergistetes*	0.06	0.09	NS	NS	0.61	-0.28	NS
*Tenericutes*	0.68	1.14	<0.1	NS	0.75	-0.42	<0.05
*TM7*	0.54	0.63	NS	NS	0.23	0.02	NS
*Verrucomicrobia*	0.52	0.53	NS	NS	0.04	-0.21	NS
*WPS-2*	0.22	0.07	NS	NS	-1.56	0.03	NS

*P-values are derived using a Wilcoxon Rank Sum test to assess the differences between treatments with FDR correction. The fold change between LRFI and HRFI bulls is also recorded in the table as is the correlation coefficient (ρ) and P-value for each phylum and RFI group.*

**Table 6:** Mean relative abundance of phyla classified in the rumen solid fraction of Simmental bulls phenotypically divergent for residual feed intake (RFI).

	Solid						
Phylum	HRFI	LRFI	*P-*value	FDR	Log2 LRF1/HRFI	ρ	*P-*value
**HRFI *n* = 13 LRFI *n* = 11**						
F:B	1.31	1.19	NS	NS	n/a	-0.42	<0.05
*Actinobacteria*	1.80	1.73	NS	NS	-0.06	0.41	<0.1
*Bacteroidetes*	31.95	33.44	NS	NS	0.07	-0.21	NS
*Chloroflexi*	0.30	0.40	NS	NS	0.45	-0.11	NS
*Cyanobacteria*	0.55	0.77	NS	NS	0.47	-0.34	<0.1
*Elusimicrobia*	0.05	0.07	NS	NS	0.41	-0.16	NS
*Euryarchaeota*	5.75	6.03	NS	NS	0.07	-0.10	NS
*Fibrobacteres*	0.91	3.42	<0.05	NS	1.92	-0.47	<0.05
*Firmicutes*	41.96	39.72	NS	NS	-0.08	0.43	<0.05
*LD1*	0.00	0.00	NS	NS	-3.27	-0.07	NS
*Lentisphaerae*	0.06	0.06	NS	NS	-0.08	-0.26	NS
*Planctomycetes*	0.17	0.39	NS	NS	1.16	-0.22	NS
*Proteobacteria*	11.90	8.49	NS	NS	-0.49	0.03	NS
*Spirochaetes*	2.28	2.57	NS	NS	0.18	-0.15	NS
*SRI*	0.01	0.01	NS	NS	0.96	0.11	NS
*Synergistetes*	0.10	0.15	NS	NS	0.66	-0.22	NS
*Tenericutes*	0.83	0.97	NS	NS	0.21	-0.27	NS
*TM7*	0.67	0.74	NS	NS	0.15	0.07	NS
*Verrucomicrobia*	0.36	0.57	NS	NS	0.69	-0.32	NS
*WPS-2*	0.09	0.09	NS	NS	-0.04	-0.16	NS

*P-values are derived using a Wilcoxon Rank Sum test to assess the differences between treatments with FDR correction. The fold change between LRFI and HRFI bulls is also recorded in the table as is the correlation coefficient (ρ) and P-value for each phylum and RFI group.*

**FIGURE 1:**
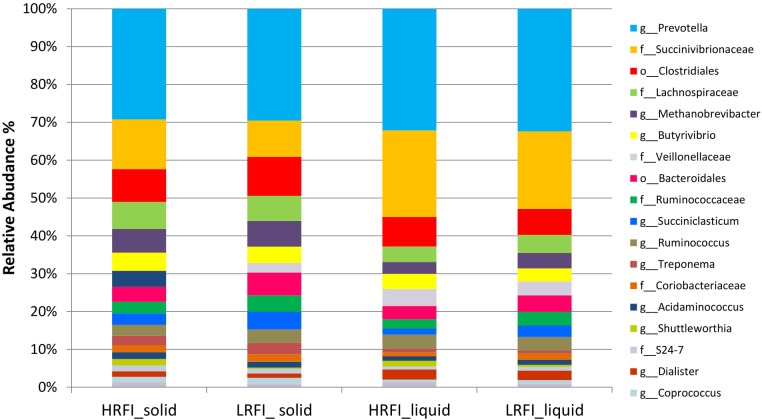
Stackplot illustrating the relative abundance of genera classified in the rumen liquid and solid fractions of Simmental bulls phenotypically divergent for residual feed intake.

At a genus level 124 genera were identified (**Figure 1**) with less than one percent of the sequences left with no taxonomic assignment.

### Relationship Between Bacterial and Archaeal Taxa and Phenotypic RFI Status

At phylum level, in the rumen liquid digesta the relative abundance of *Cyanobacteria, Tenericutes* and *Fibrobacteres* were negatively correlated with RFI (*P* < 0.05) (**Table 5**). In the rumen solid digesta the relative abundance of *Fibrobacteres* (*P* < 0.05) and *Cyanobacteria* (*P* < 0.1) were negatively correlated with RFI. It was also observed that F:B ratio was positively correlated with RFI in the solid ruminal phase (*P* < 0.05) (**Table 6**).

**FIGURE 2:**
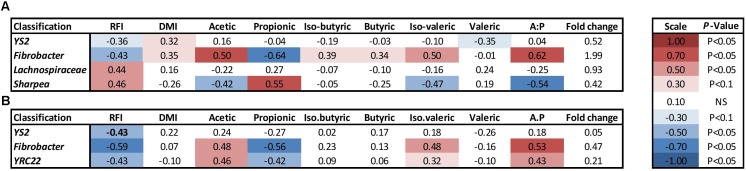
Illustration of the relationship between rumen genera and phenotypic RFI, DMI and relative proportions of residual VFA present in the rumen. **(A)** solid ruminal phase and **(B)** liquid ruminal phase. Spearman’s correlation coefficient was used to deduce the relationships.

**FIGURE 3:**
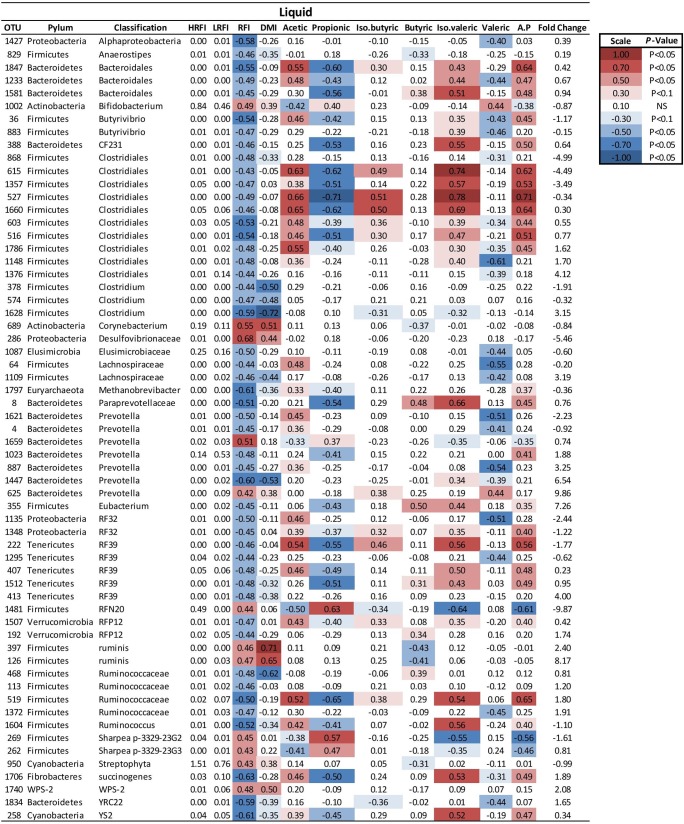
Mean relative abundance of OTUs present in the liquid ruminal phase and their relationship and phenotypic RFI, DMI and relative proportions of residual VFA present in the rumen. Spearman’s correlation coefficient was used to deduce the relationships.

**FIGURE 4:**
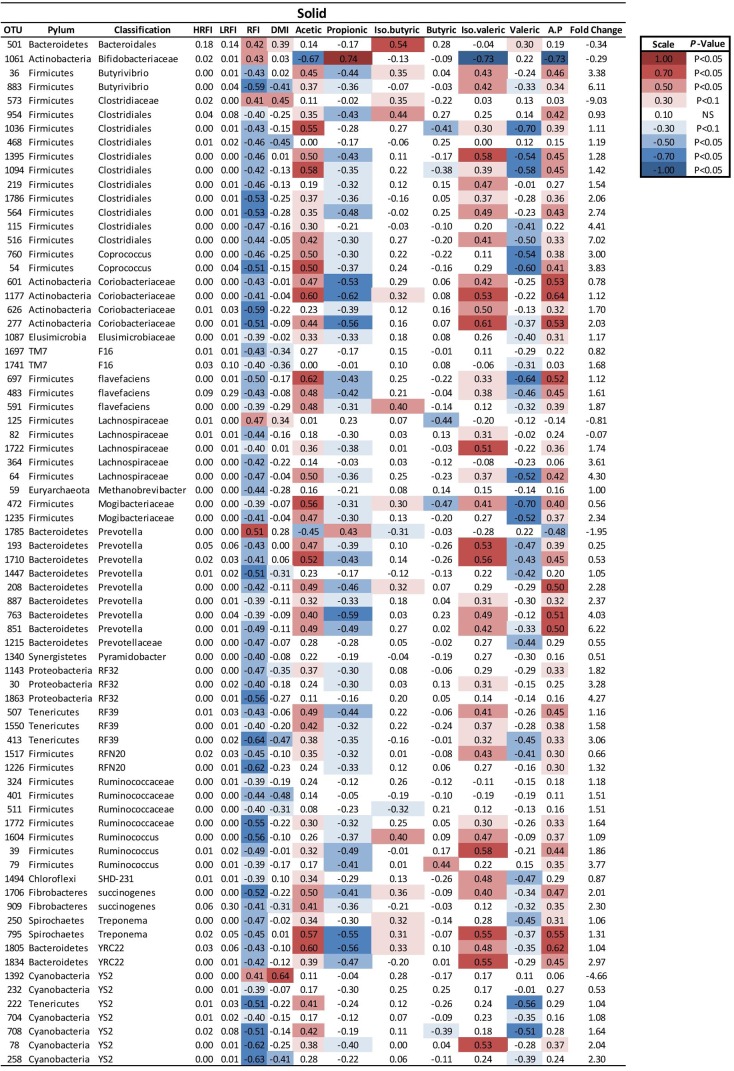
Mean relative abundance of OTUs present in the solid ruminal phase and their relationship and phenotypic RFI, DMI and relative proportions of residual VFA present in the rumen. Spearman’s correlation coefficient was used to deduce the relationships.

At a genus level in the solid ruminal fraction there was a positive relationship between the relative abundance of *Sharpea* and unclassified genera from the *Lachnospiraceae* and RFI (*P* < 0.05) (**Figure 1**). The relative abundance of *Fibrobacter* and *YS2* (*Cyanobacteria*) were negatively correlated (*P* < 0.05) with RFI. In the liquid rumen fraction, a negative relationship between the relative abundance of *Fibrobacter*, *YRC22 (Paraprevotellaceae)* and *YS2* (*Cyanobacteria*) and RFI was observed (*P* < 0.05) (**Figure 2**). OTUs exhibiting a significant relationship with RFI in the solid and liquid fraction were summarized in **Figures 2**, **3**, respectively. There were some common relationships observed between OTUs present in the solid and liquid ruminal fractions and RFI. OTUs assigned as *Butyrivibrio, Clostridales, Elusimicrobiaceae, Fibrobacter succinogenes*, *Methanobrevibacter, Unclassified RF32, Unclassified RF39*, *Ruminococcaceae*, *Ruminococcus*, *Unclassified YS2* (*Cyanobacteria*), *and Unclassified YRC22 (Paraprevotellaceae)* were negatively correlated with RFI in both solid and liquid fractions (*P* < 0.05). A positive correlation was observed for OTUs assigned as *Bifidobacteriaceae* and RFI in both the solid and liquid phase (*P* < 0.05) (**Figures 3**, **4**).

Two *Methanobrevibacter* OTUs, OTU 1797, and OTU 58 were correlated with RFI in the solid and liquid ruminal phase, respectively. These were identified to closely match *Methanobrevibacter millerae* YE315 (97%) and *Methanobrevibacter* ABM4 (99%) in the solid and liquid phase, respectively.

### Relationship With *Fibrobacter*

*Fibrobacter succinogenes* showed a negative correlation with RFI abundance which was apparent from species to phylum level. In the liquid digesta, there were 39 genera that were positively correlated with the relative abundance of *Fibrobacter* whereas 12 genera were negatively correlated with the relative abundance of *Fibrobacter* (*P* < 0.05). In the solid digesta, there were 39 genera that were positively correlated with the relative abundance of *Fibrobacter* whereas 22 genera were negatively correlated with the relative abundance of *Fibrobacter* (*P* < 0.05). There were 38 consistent relationships linking *Fibrobacter* and other genera present in the rumen between solid and liquid digesta (*P* < 0.05). Results of these relationships are summarized in **Table 7**.

**Table 7:** Relationships between relative abundance of *Fibrobacter* and other genera present in the rumen solid and liquid digesta.

Phylum	Classification	Liquid ρ	*P-*value	Solid ρ	*P-*value
*Proteobacteria*	*GMD14H09*	0.48	0.01	0.85	0.00
*Firmicutes*	*Ruminococcus*	0.68	0.00	0.79	0.00
*Bacteroidetes*	*Bacteroidales*	0.56	0.00	0.76	0.00
*Firmicutes*	*RFN20*	0.45	0.02	0.70	0.00
*Firmicutes*	*Ruminococcaceae*	0.62	0.00	0.68	0.00
*Bacteroidetes*	*RF16*	0.66	0.00	0.67	0.00
*Euryarchaeota*	*vadinCA11*	0.52	0.01	0.65	0.00
*Firmicutes*	*Selenomonas*	0.40	0.04	0.64	0.00
*Firmicutes*	*Clostridiaceae*	0.47	0.01	0.64	0.00
*Synergistetes*	*Pyramidobacter*	0.72	0.00	0.63	0.00
*Firmicutes*	*Clostridium*	0.50	0.01	0.62	0.00
*Bacteroidetes*	*CF231*	0.51	0.01	0.60	0.00
*Bacteroidetes*	*YRC22*	0.63	0.00	0.60	0.00
*Actinobacteria*	*Adlercreutzia*	0.53	0.00	0.59	0.00
*Bacteroidetes*	*BF311*	0.40	0.04	0.59	0.00
*Firmicutes*	*Anaerovibrio*	0.46	0.02	0.59	0.00
*Tenericutes*	*Anaeroplasma*	0.61	0.00	0.58	0.00
*Firmicutes*	*Pseudobutyrivibrio*	0.44	0.02	0.58	0.00
*Planctomycetes*	*Pirellulaceae*	0.52	0.01	0.56	0.01
*Spirochaetes*	*Spirochaetaceae*	0.49	0.01	0.54	0.01
*Firmicutes*	*Anaerofustis*	0.52	0.01	0.54	0.01
*Actinobacteria*	*Atopobium*	0.49	0.01	0.53	0.01
*Euryarchaeota*	*Methanobrevibacter*	0.57	0.00	0.53	0.01
*Chloroflexi*	*SHD231*	0.57	0.00	0.53	0.01
*Firmicutes*	*Mogibacterium*	0.74	0.00	0.51	0.01
*Firmicutes*	*L7A*	0.52	0.01	0.50	0.01
*Firmicutes*	*Moryella*	0.56	0.00	0.48	0.02
*Proteobacteria*	*Sutterella*	-0.39	0.04	-0.48	0.02
*Bacteroidetes*	*Prevotella*	-0.44	0.02	-0.49	0.02
*Firmicutes*	*Acidaminococcus*	-0.44	0.02	-0.50	0.01
*Firmicutes*	*Bulleidia*	-0.48	0.01	-0.54	0.01
*Firmicutes*	*Catenibacterium*	-0.53	0.00	-0.60	0.00
*Firmicutes*	*Veillonellaceae*	-0.42	0.03	-0.66	0.00
*Firmicutes*	*Mitsuokella*	-0.39	0.05	-0.67	0.00
*Actinobacteria*	*Bifidobacteriaceae*	-0.62	0.00	-0.69	0.00
*Firmicutes*	*Lachnospiraceae*	-0.63	0.00	-0.77	0.00
*Firmicutes*	*Shuttleworthia*	-0.52	0.01	-0.78	0.00
*Firmicutes*	*Roseburia*	-0.55	0.00	-0.87	0.00

*Only consistent relationships seen in both the solid and liquid digesta are summarized in the table. Spearman’s correlation coefficient was used to deduce the relationships.*

## Discussion

This study examined the effect of RFI phenotype on ruminal bacteria and archaeal populations and VFA profiles of Simmental bulls offered an *ad libitum* concentrate diet. Analysis of population diversity in solid and liquid phases of the rumen digesta showed that these fractions contained similar community profiles. This is consistent with a study from Ji et al. (2017), which discerned similar bacterial diversity from unseparated rumen digesta in comparison to rumen samples fractionally separated into liquid and solid via cheese cloth. Schären et al. (2017) also found a high level of similarity between the solid and liquid ruminal fractions, despite a more stringent separation technique. This is likely due to the homogenous nature of the two fractions (Sadet et al., 2007). In order obtain a stringent separation of solid and liquid fractions require separation steps from which nucleic acid degradation could potentially ensue. Nucleic acid integrity was a priority for this experiment therefore; simple but efficient separation via cheesecloth was utilized (Fliegerova et al., 2014).

*Prevotella* and an uncultured *Proteobacteria* genus of bacteria, identified as a member of the *Succinivibrionaceae* family, were the dominant genera in both liquid and solid digesta present in the LRFI and HRFI animals of the current study. This is supported by global studies (Henderson et al., 2015) where these genera were found to be more abundant in cattle fed high-energy concentrate diets (Henderson et al., 2015). *Prevotella* and unclassified *Succinivibrionaceae* are the major producers of propionate and succinate in the rumen of animals fed concentrate rich diets (Murphy et al., 1982; Chen et al., 2017). Succinate does not accumulate in the rumen and is rapidly converted to propionate (Suen et al., 2011), which is transported out of the rumen via the rumen wall and converted to glucose through the gluconeogenesis pathway (McDonald et al., 2002). The high relative abundance of *Prevotella* and unclassified *Succinivibrionaceae* imply that the divergent cohorts should both be producing a relatively high concentration of propionate, thus, the lower molar proportion of propionate in the rumen of LRFI animals suggest alternative fermentative or absorptive patterns in these animals. Low propionate concentrations have previously been associated with feed inefficient cattle (Krueger et al., 2008). It has been suggested that inter-animal variation in ruminal VFA concentrations may contribute to some of the observed variation in RFI (Guan et al., 2008; Krueger et al., 2009; McDonnell et al., 2016; Shabat et al., 2016), however, to date there has been a lack of consistency in the results of VFA profiles of cattle divergent for feed efficiency (Guan et al., 2008; Hernandez-Sanabria et al., 2010; Fitzsimons et al., 2013; McDonnell et al., 2016; Shabat et al., 2016). There was an increase in A:P ratio in LRFI in comparison to HRFI bulls, this can be attributed to the reduced molar proportions of propionate in the rumen of LRFI animals and consistent molar proportions of acetate between LRFI and HRFI animals. A higher ruminal A:P ratio in LRFI heifers was previously observed by Krueger et al. (2009).

The phylum of *Fibrobacteres* showed a negative relationship with RFI in both ruminal fractions as did OTUs assigned as *F. succinogenes*. *F. succinogenes* is known for its importance as an efficient and prolific degrader of cellulose in the rumen, which produces three major end products; succinate, acetate, and formate (Miller, 1978). Unlike other rumen bacteria which derive energy from numerous polysaccharide sources, *F. succinogenes* is specialized only for the utilization of cellulose as a substrate (Suen et al., 2011). The genome of *F. succinogenes* encodes numerous enzymes capable of degrading a wide array of polysaccharides and it has been suggested it uses these enzymes to remove plant cell wall hemicelluloses in order to gain access to cellulose (Suen et al., 2011). It is hypothesized that these hemicellulose breakdown products provide growth substrates for the other bacteria present in the rumen (Suen et al., 2011) which is supported by our data by the strong relationships observed between the relative abundance of *Fibrobacter* and other genera present in the rumen. As mentioned above, succinate does not accumulate in the rumen, the decarboxylation of succinate, which is produced by *F. succinogenes*, is predicted to be facilitated by *Selenomonas ruminantium* in the rumen (Suen et al., 2011), which exhibits a positive relationship with *F. succinogenes* in the current study. The 16S rRNA results indicate that there is potentially an increase in the cellulolytic activity in the rumen of LRFI bulls and to further add evidence to this we note a positive correlation between *F. succinogenes* with acetate which is one of the main products of *F. succinogenes’s* metabolic pathway. It is proposed that the LRFI animals have a higher abundance of *F. succinogenes* and that the succinate produced by this bacterium is rapidly converted to propionate by *S. ruminantium* thereby providing a higher level of substrate available for gluconeogenesis. However, this hypothesis was based on 16S DNA, which may not represent metabolically active species and therefore will require further investigation.

Increased rumen butyrate has been associated with greater feed efficiency in cattle (Guan et al., 2008; Shabat et al., 2016) and the findings of the current study are in agreement with this result, with a higher concentration of butyric acid tending to be observed for the LRFI bulls. Correspondingly we noted increased in plasma βHB in LRFI animals. Butyric acid has been associated with increased mitotic activity in the rumen epithelial cells (Sakata and Tamate, 1978). In the rumen epithelium, butyrate is converted to βHB, which is an important energy source for many host tissues (McDonald et al., 2002). Efficient cattle have been shown to have increased rumen epithelium thickness compared with inefficient contemporaries (Lam et al., 2017). It is possible that greater butyrate availability in the rumen of LRFI animals may increase the rumen surface area for absorption, therefore proliferating absorption of high energy VFAs, such as propionate from the rumen to the liver (Daniel and de Resende Júnior, 2012; Keogh et al., 2017). Decreased absorption of VFAs across the rumen epithelium has been previously associated with low feed efficiency (Albornoz et al., 2013; Zhang et al., 2013). In addition, research from our own group has provided evidence of up regulation in transcriptional activity in the rumen epithelium of feed efficient cattle, suggesting a higher rate of nutrient absorption under this feeding regime (Keogh et al., 2017).

OTUs identified as *Butyrivibrio*, a butyrate producing genera, were negatively correlated with RFI in both the solid and liquid digesta. Myer et al. (2015) had previously identified a higher abundance of *Butyrivibrio* in efficient steers. *Butyrivibrio* are members of the *Lachnospiraceae* family. OTUs assigned to *Coprococcus*, another butyrate producing genus from the *Lachnospiraceae*, showed a negative relationship with RFI in the rumen liquid digesta in the current study. This association has been previously observed in the rumen of LRFI cattle (Jewell et al., 2015). Shabat et al. (2016) noted an enrichment of *Coprococcus* and the metabolic pathways it encodes in LRFI dairy cows. Increased abundance of *Lachnospiraceae* is noted in HRFI animals in the current study at genus level. In our data, OTUs only assigned as far as *Lachnospiraceae* exhibited both a positive and negative relationship with RFI. *Lachnospiraceae* has been positively correlated with feed efficiency (Rius et al., 2012; Shabat et al., 2016), however, certain members of this family have also been found to be more abundant in inefficient animals in the current study and in the literature (Shabat et al., 2016; Li and Guan, 2017). *Ruminococcaceae* and *Ruminococcus* OTUs were negatively correlated with RFI, indicative of a role in enhancing the efficiency in LRFI bulls. OTUs assigned as *Ruminococcus flavefaciens* held a negative relationship with RFI. *R. flavefaciens* are an active cellulolytic bacterial species which are succinate producers, again suggestive of alternate fermentation patterns in the rumen of LRFI bulls (Pettipher and Latham, 1979).

The phylum of *Cyanobacteria* exhibited a negative relationship with RFI. The OTUs driving this relationship were from the order of *YS2.* The *YS2* order has previously been found in the ruminant gut (AlZahal et al., 2017; Neves et al., 2017) and has many functions, including obligate anaerobic fermentation, nitrogen fixation, syntrophic hydrogen production, and synthesis of vitamin B and K synthesis (Di Rienzi et al., 2013). The phylum of *Cyanobacteria* is known for its photosynthetic capability but a new candidate phylum has been suggested, “*Melainabacteria*” which would encompasses non-photosynthetic members currently assigned to this phylum, inclusive of *YS2* (Di Rienzi et al., 2013). Despite *YS2* being previously identified in cattle (AlZahal et al., 2017; Neves et al., 2017), no previous association with feed efficiency has been recorded. The positive correlation between RFI and *YS2* observed in the current study may indicate a possible niche role for *YS2* in nutrient supply (Zeng et al., 2015) in LRFI bulls.

*Tenericutes* was also negatively correlated with RFI, OTUs within this phylum, including *RF39*, were also negatively correlated with RFI. This contradicts Jami et al. (2014) who identified a positive tentative correlation between RFI and *RF39* in dairy cows, while in humans lean BMI subjects were enriched with *RF39* (Goodrich et al., 2014), suggesting humans with reduced energy harvest capacity harbor *RF39*.

*YRC22* from the *Paraprevotellaceae* family showed a negative relationship with RFI as did OTUs assigned as *YRC22*. *YRC22* has previously been shown to be more abundant in the rumen solid fraction of low RFI dairy cattle (Jewell et al., 2015) and in dairy cattle supplemented with tannins and are therefore deemed as more efficient than their supplemented controls (Díaz Carrasco et al., 2017). Myer et al. (2015) found no difference in abundance of *YRC22* steers divergent for feed efficiency, however, an alternate measure of feed efficiency was utilized by Myer et al. (2015) classifying animals based on their feed intake and growth measurement, which may have led to confounding results. The literature indicates no conclusive result on the relationship between *YRC22* and feed efficiency in cattle, however, *Paraprevotellaceae* has been found to be more abundant in obese rats, indicating this family may facilitate increased energy harvest in the rat gut (Hakkak et al., 2017).

Residual feed intake is also associated with the lactate producing genera with OTUs annotated as *Sharpea p-3329-23G2* and *p-3329-23G3* showing a positive relationship with RFI. Jewell et al. (2015) found an increase in abundance of *Sharpea* OTUs in HRFI dairy cows complementing the results of the current study. Additionally *Sharpea* is from the *Erysipelotrichaceae* family the abundance of which was reduced in LRFI porcine feces in comparison to HRFI (McCormack and Curiao, 2017). This family has also been associated with inflammation in the human gut (Kaakoush, 2015) indicating possible links with inefficiency and host inflammation.

*Methanobrevibacter millerae YE315* is negatively correlated with RFI in the rumen fluid. This is a hydrogenotrophic species which is a member of the gottschalkii clade, and encodes only one version of the methyl coenzyme reductase (*MCR*) isoenzyme, *mcr I* (Li, 2016). This indicates it thrives in a low hydrogen environment (Reeve et al., 1997). Our group has previously observed a negative relationship between this genera and RFI in both Charolais and Holstein Friesian steers fed a grain based diet (McGovern et al., unpublished). The relative abundance of *Fibrobacter* is linked with that of *Methanobrevibacter*. It is possible that *Fibrobacter* directly influences proliferation of species within these genera through the production of hydrogenotrophic substrate formate or indirectly (Suen et al., 2011); through providing substrate to other species whose fermentation end products are substrate for members of *Methanobrevibacter* genera. This relationship is also evident with another methanogenic genera; *vadinCA11* from the *Methanomassiliicoccaceae* family. This family is obligate H_2_-dependent methylotrophs, utilizing methyl groups from methanol and methylamines (mono-, di-, and tri-methylamine) and methyl thiols for methanogenesis (Lambie et al., 2015). It is probable here that *Fibrobacter* indirectly provides substrates (Suen et al., 2011) to *Methanomassiliicoccaceae.* For example, *Succinivibrionaceae* degrade pectin a structural polysaccharide contained in certain plant cell wall to produce methanol (O’Herrin and Kenealy, 1993), which is a substrate required for the growth of *Methanomassiliicoccaceae* (Paul et al., 2012). Further functional analysis will be required to investigate the proposed relationships from these data.

In agreement with the hypothesis that LRFI phenotype is potentially related to increased capability of rumen microbiota to harvest nutrients from ingested feed, in the solid ruminal digesta phase F:B is negatively correlated with RFI. This indicates that an increased F:B ratio may support a more feed efficient animal. In dairy cattle F:B ratio has been associated with increased milk fat-yield (Jami et al., 2014). This ratio has also been used in human and murine studies with increased F:B ratio an indication of obesity (Ley et al., 2005; Turnbaugh et al., 2006) with Ley et al. (2006) suggesting that in obese mice the higher ratio was due to an effective release of energy through digestion. In a recent study Shabat et al. (2016) suggested efficient dairy cattle had a less diverse microbial community that produced relevant metabolites to promote growth. In the human gut a low diversity gut microbiome has been associated with obesity (Consortium, 2012). In relation to animal protein production, a low diversity microbial population in the rumen focused on promoting energy yield from feed is desirable (Shabat et al., 2016). However, there was no evidence of divergence in microbial diversity between cattle divergent for RFI in the current study.

In conclusion this study examined the effect of RFI phenotype on ruminal bacterial and archaeal populations present in the rumen of Simmental bulls offered a concentrate based finishing diet. This study suggests that RFI phenotype does not affect the overall diversity of the bacterial or archaeal communities present in the rumen of beef cattle. However, it also compliments previous research indicating that microbiota with high energy harvesting capacities are required for feed efficient weight gain with a relationship evident between F:B ratio and feed efficiency. The majority of the taxa identified as exhibiting a relationship between in their relative abundance with host feed efficiency were recognized to possess potent cellulolytic capacities or had previously been noted to cause metabolic shifts in their host. The data indicate that there is an increase in residual butyrate in the rumen of LRFI bulls which corresponded to an increase in βHB, perhaps suggestive of increased epithelial absorption. *Fibrobacter succinogenes* showed a negative relationship with RFI which was apparent from species to phylum level. The unique cellulolytic metabolism of *Fibrobacter succinogenes* suggests the bacterium may have contributed to host efficiency by providing substrates to other microbial population in the rumen. We provide evidence that abundance of certain bacterial genera and bacterial and archaeal OTUs exhibit relationships with RFI phenotype, therefore, phenotypic expression of RFI may potentially be influenced through the role of bacterial and archaeal in ruminal degradation of complex plant polysaccharides. The data also suggest that it is probable that microbial species or strains may impact the efficiency of the animal more than an overall ruminal community shift. Therefore, in order to investigate phenotypic impact of RFI on the microbiome, global high depth metatranscriptomic sequencing is required to deduce this complex symbiotic relationship.

## Author Contributions

DK, MM, AK, and SW conceived and designed the experiments. EM, MSM, and CF performed the experiments. EM, MSM, and CF analyzed the data. SW, DK, MM, AK, SW, MSM, and CF contributed reagents, materials, and analysis tools. EM, SW, DK, MG, AK, MSM, MM, and CF result interpretation and wrote paper.

## Conflict of Interest Statement

The authors declare that the research was conducted in the absence of any commercial or financial relationships that could be construed as a potential conflict of interest.

## Footnotes

1 https://sourceforge.net/projects/bbmap/
