# Supplementary material for: 16S rRNA Sequencing Reveals Relationship Between Potent Cellulolytic Genera and Feed Efficiency in the Rumen of Bulls
Source: Front Microbiol. 2018 Aug 10;9:1842. doi: 10.3389/fmicb.2018.01842 (PMC6097346; doi:10.3389/fmicb.2018.01842)
Supplement: Supplementary file 1 [file Image_1.pdf]

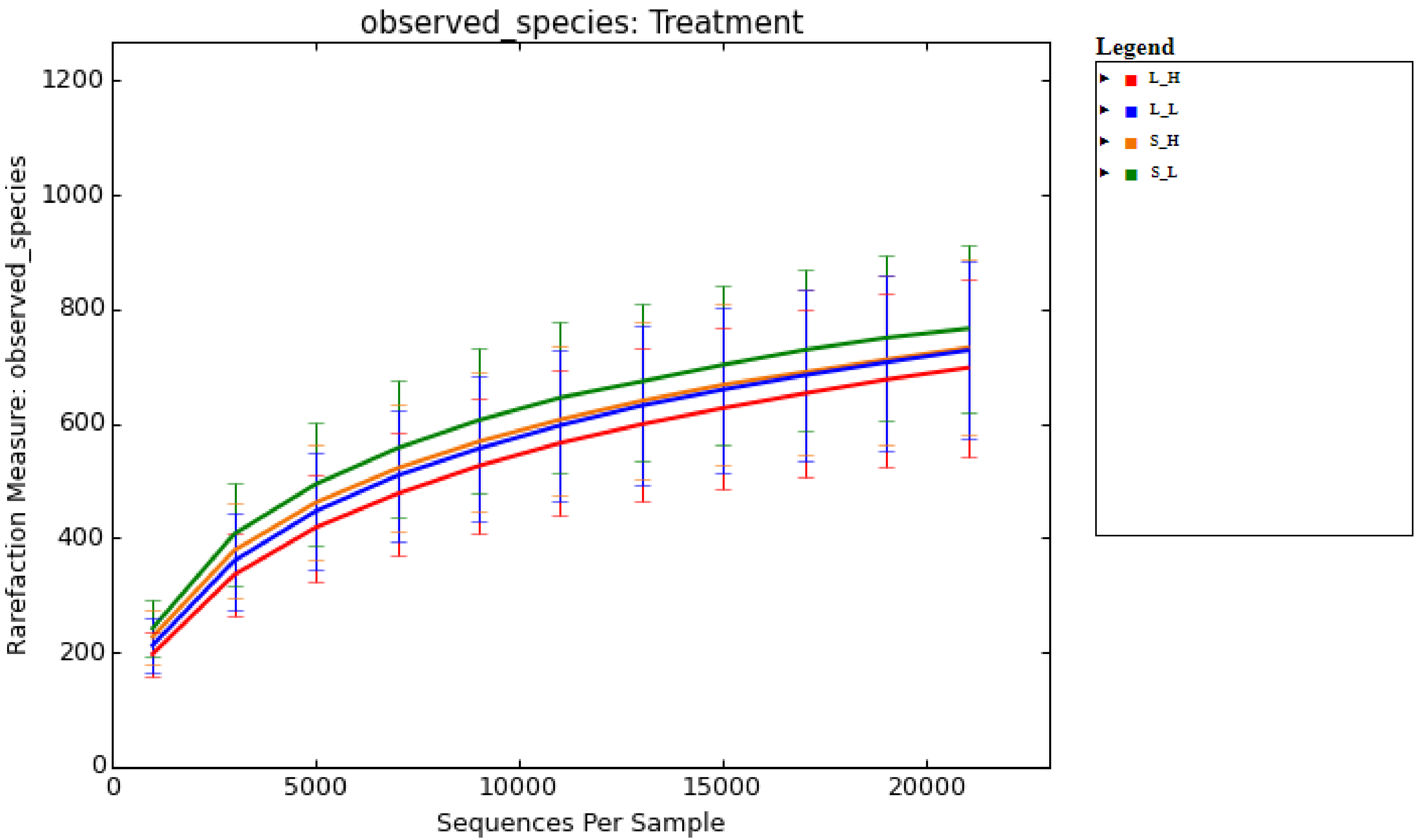


**Supplementary Figure S1.** Rarefaction curves of observed species (i.e., OTUs) clustered at the 97% identity based on the 16S rRNA gene sequences of rumen solid (S) and liquid (L) digesta of Simmental bulls phenotypically divergent for residual feed intake. High RFI = H, Low RFI = L
